# General Practitioners as partners for a shared management of chronic HIV infection: An insight into the perspectives of Italian People Living with HIV

**DOI:** 10.1371/journal.pone.0254404

**Published:** 2021-07-09

**Authors:** Serena Rita Bruno, Mariacristina Poliseno, Francesca Vichi, Sara Esperti, Antonio Di Biagio, Marco Berruti, Sergio Ferrara, Luigi Pisani, Annalisa Saracino, Teresa Antonia Santantonio, Sergio Lo Caputo

**Affiliations:** 1 Department of Clinical and Experimental Medicine, Infectious Diseases Unit, A.O.U. “Policlinico Riuniti”, Foggia, Italy; 2 Department of Biomedical Sciences and Human Oncology, Clinic of Infectious Diseases, University of Bari “Aldo Moro”, Bari, Italy; 3 Unit of Infectious Diseases, Santa Maria Annunziata Hospital—Bagno a Ripoli, Florence, Italy; 4 Department of Health Sciences, Unit of Infectious Diseases, San Martino Policlinico Hospital, University of Genoa, Genoa, Italy; 5 Department of Intensive Care, Amsterdam University Medical Centers—Location AMC, Amsterdam, The Netherlands; Universita degli Studi della Campania Luigi Vanvitelli, ITALY

## Abstract

Is it possible to achieve a collaboration between Infectious Diseases (ID) Specialists and General Practitioners (GPs) in the management of chronic HIV infection? A cross sectional survey was conducted among People Living with HIV (PLWHIV) attending the outpatient services of four Italian Infectious Diseases Centers to understand to which extent patients trust their GPs and involve them in the management of their chronic condition. Information about level of communication with GPs, subjective perception of the disease, and presence of co-medications were collected and matched with socio-demographic data using χ^2^statistics. A *p<0*.*05* was considered statistically significant. From December 2019 to February 2020, 672 patients completed the survey, 59% males and 56% >50 years. Overall, 508 patients (76%) had informed GPs about HIV-positivity. Communication of diagnosis was significantly associated with age >50years, lower education level, history of disease >10 years and residency in Northern Italy. The “Undetectable = Untrasmittable” (U = U) concept was investigated as an indirect measure of perceived stigma. 23% of subjects was unaware of its meaning. Despite undetectable status, 50% of PLWHIV found difficult to communicate their condition to GPs, especially married (52% *vs* 48% of unmarried, *p = 0*.*003)*, well-educated patients (51% *vs* 48, *p = 0*.*007)*, living in Southern *vs* Northern Italy (52% *vs* 46%, *p< 0*.*001*). More than 75% of the participants consulted the ID specialist for co-medications and DDIs management, often complaining a lack of communication of the former with GPs. Overall, a good level of communication between PLWHIV and GPs was outlined, even if a wider involvement of the latter in HIV care is desirable.

## Introduction

In the modern combined Antiretroviral Therapies (cART) era, life expectancy for patients with chronic HIV infection has remarkably increased and People Living with HIV (PLWHIV) can now be considered as an ageing cohort, with health issues that resembles those of the general population [1–3].

Historically, the care of HIV positive individuals has been provided in HIV specialist clinics and currently, at least in Italy, the management of chronic HIV infection is almost totally hospital-centered with limited involvement of other health-care figures.

As a consequence, Infectious Diseases (ID) Specialists today are called to embrace challenges that exceed their usual field of expertise, as limiting chronic cART toxicities [4,5], managing poly pharmaceutical therapies and drug-drug interactions (DDIs) [6], facing ageing-related health issues [7,8] and, ultimately, providing correct scientific information to patients who often surf the Internet in search of health information and get easily caught in fake news and misleading information regarding their chronic condition [9].

New partners should urgently be found to build up a shared, multi- disciplinary management of PLWHIV aimed to improve the quality of care and to reduce the pressure on ID Clinics through the decentralization of the treatment process.

General Practitioners (GPs) embody ideal candidates for this task, and examples of collaboration between GPs and Specialists in the management of HIV positive patients have already been proposed [10–12].

On the other hand, understanding if PLWHIV are willing to share their care pathway with GPs or with other health care figures is fundamental as the role of patient’s participation in the care process is crucial to increase compliance, treatment adherence, and thereby to improve the whole health outcome [13].

Remarkably, only a few works have focused on this topic so far [14–16] and none of them was performed in Italy.

Aim of this survey was to collect the perspectives of a cohort of Italian PLWHIV on the relationship with their GPs and to analyze multiple factors that could have an influence on this process.

## Material and methods

We conducted an interview- study by distributing an anonymous questionnaire among outpatients attending for lab tests and/or medical visit in four major Italian Hospitals, both teaching (University) and non- teaching. The study was performed in the Units of Infectious Diseases of Bari and Foggia University Hospitals, in the Unit of Infectious Diseases of Santa Maria Annunziata Hospital in Florence and in the Unit of Infectious Diseases of San Martino University in Genoa.

The questionnaire included 16 items organized into two main sections (S1 and S2 Tables).

Questions 1 to 7 collected socio-demographic information (sex, school education, town of residence, age, marital status, housing situation, time from HIV diagnosis); answers in this part where descriptive and multiple-choice with three/four possibilities.

Questions 8 to 16 were formulated with lean answers (*yes/no/I don’t know*) and investigated three main topics: i) communication of HIV positivity to GPs, ii) patient’s subjective perception of the disease with special attention to the concept of U = U, iii) management of Drug-Drug Interactions (DDIs) and comedications, when present.

**Fig 1:**
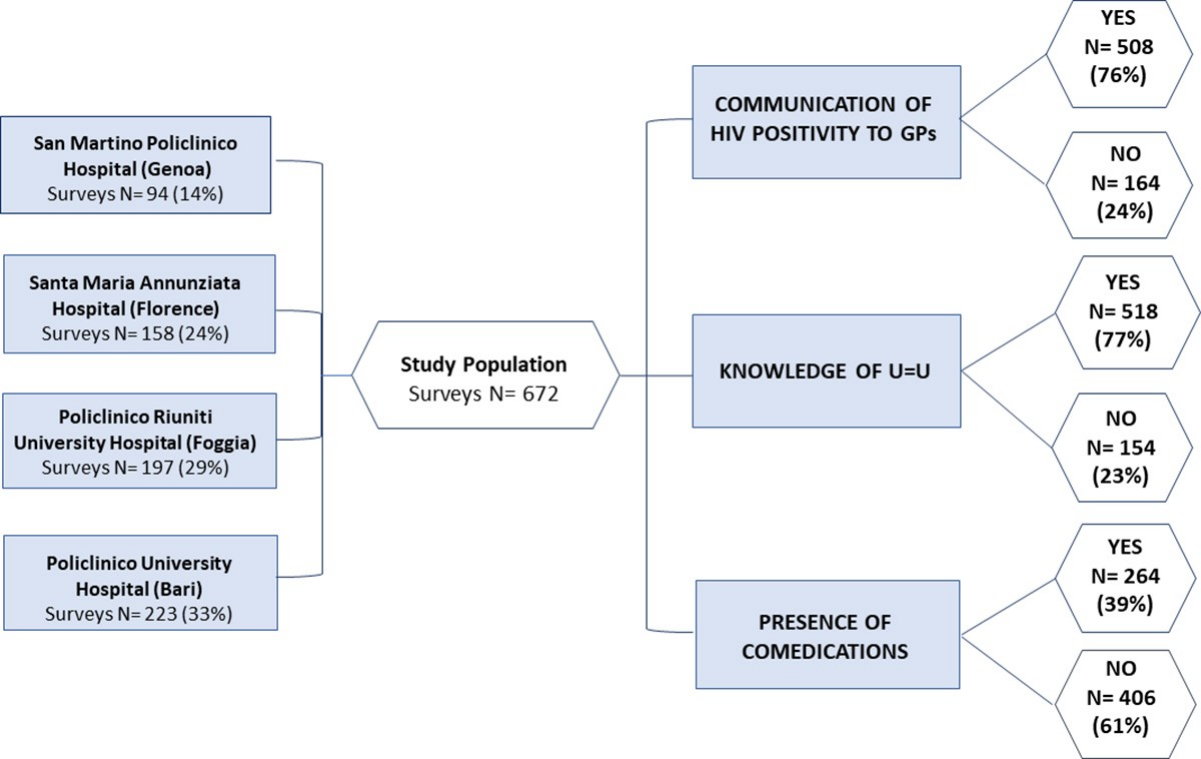
Study design. The number of surveys collected in the four participating Centers is reported, along with three main topics investigated (communication of HIV positivity to GP, knowledge of U = U, presence of comedications) and frequency of answers collected for each topic.

Study design is reported in Fig 1 as PRISMA flow-chart.

The survey tool was self-designed by our working group and all co-authors independently assessed its content validity. A pilot version of the questionnaire was preliminary performed on a small subset of participants (30 patients for each Centre) by two independent researchers in Foggia and Bari University Hospital respectively. Researchers personally performed accompanied- interviews to assess the face validity of the survey and to outline and eliminate possible confounding elements.

The final version of the questionnaire was formulated in Italian language and was anonymous.

Medical and nursing staff were involved in delivering the survey and in providing verbal information about its content, but patients self-compiled the answers, so that their identities and feedbacks were impossible to trace back.

Participation was completely voluntary: no formal written consent was requested, as consent was implied by the completion of the questionnaire. Patients were assured that their answers were confidential. The Ethical Committee of the Coordinating Centre (Foggia University Hospital) was consulted for ethical approval: in consideration of the specific study design, ethical approval was deemed not necessary.

Patients were enrolled at the condition of: being Italian, adult (≥18 years old), diagnosed with HIV infection from at least one year, assuming cART from at least six months. The latter two questions were asked by the staff before the completion of the survey, that was interrupted in case of negative response.

Questionnaires distribution was planned between 1^st^ December 2019 and 31^st^ May 2020.

A recruitment target of 1000 patients was set, considering saturation reached when each Unit participating the survey would have provided approximatively 250 questionnaires.

Unfortunately, data collection was stopped at the beginning of February 2020, as a consequence of the discontinuation of the out-patients services in all the country started with the explosion of Sars-COV-2 pandemic and still going on; therefore, herein we present a preliminary data analysis.

Rates of attendance of the indicate that, overall, more than 3000 potentially eligible service users are followed in the four participating Centres, attending visit and/or laboratory tests with a frequency of at least four times per year: about 500 patients per Centre in the two-months period considered. The number of questionnaires collected suggests that approximatively the 30% of eligible patients has completed and returned the questionnaire.

## Statistics

Socio demographic data (answers to questions 1–6) were divided into categorical variables and descriptive statistics was performed in terms of absolute frequencies (percentage).

Pearson Χ^2^ test was used to compare the answers given to questions 7 to 15 (regarding the communication of HIV positivity, the subjective perception of the disease and the presence and management of co medications) with categorical variables (residence, gender, level of education, duration of disease, age, marital status) with the aim of outline any feature that could possibly have an influence on patient’s relationship with GPs.

Analysis was performed using R v.3.60. A *p<0*.*05* was considered statistically significant.

## Results

During the observation period, 672 patients completed the survey.

Socio-demographic characteristics of the study population divided into categorical variables are reported in **Table 1**.

**Table 1:** Socio-demographic characteristics of 672 PLWHIV participating the survey.

Patients features	N (%)
**Sex**	
*Males*	462 (69)
*Females*	210 (31)
**Age**	
*>50 years*	379 (56)
*<50 years*	293 (44)
**Education level**	
Elementary license or inferior	285 (42)
High school license or superior	387 (58)
**Residence**	
Northern Italy*	246 (36)
Southern Italy**	426 (64)
**Marital status**	
Married	241 (35)
Not Married	431 (65)
**Housing situation**	
Living with spouse/children	241 (35)
Living alone	200 (29)
Living with parents	107 (16)
Living with friends/partners	124 (18)
**Time from diagnosis**	
<10 years	337 (50)
>10 years	335 (50)

* Northern Italy: Florence, Genoa

** Southern Italy: Bari, Foggia.

### Communication of HIV positivity to GPs

Among the participants, 76% (n = 508) had informed the GP about HIV-positivity status.

164 patients affirmed to have not informed their GP of their HIV diagnosis, of whom 64 (39%) because of concern about data protection, 61 (37%) due to fear of stigma; lastly,39 patients (24%) considered communicating their serological status to the GPs not necessary for their routine general health management.

Overall, 116/164 patients (70%) reported that, if none of the previous mentioned concerns existed, they would find beneficial to share their information about their chronic infective condition with their physicians.

At univariate analysis, socio-demographic variables associated with a better communication with GPs were age >50 years, low level of education, residency in Northern Italy, as reported in **Table 2**. No differences were reported for sex or marital status.

**Table 2:** Communication of HIV positivity to GPs according to socio-demographic features.

	*“Yes”*	*“No”*	*p value*
**Sex**			
Males (N = 462)	341 (74%)	121 (26%)	*p = 0*.*133*
Females (N = 210)	167 (79%)	43 (21%)	
**Age**			
<50years (N = 293)	182 (62%)	111 (38%)	***p< 0*.*001***
>50years (N = 379)	326 (86%)	53 (14%)	
**Education level**			
Elementary license or inferior (N = 285)	240 (84%)	45 (16%)	***p< 0*.*001***
High school license or superior (N = 387)	268 (69%)	119 (31%)	
**Geographical position**			
Northern Italy* (N = 246)	212 (86%)	34 (14%)	***p<0*.*001***
Southern Italy** (N = 426)	296 (70%)	130 (30%)	
**Marital status**			
Married (N = 241)	182 (75%)	59 (25%)	*p = 0*.*782*
Not married (N = 431)	326 (76%)	105 (24%)	
**Time from diagnosis**			
<10years (N = 337)	237 (70%)	100 (30%)	***p = 0*.*001***
>10years (N = 335)	**271 (81%)**	64 (19%)	

*Northern Italy: Florence, Genoa

** Southern Italy = Bari, Foggia.

### Patient’s subjective perception of the disease

The concept of “*Undetectable = Untrasmittable*” (U = U) was investigated and taken as an indirect measure of perceived stigma.

To note, 154 (23%) subjects out of 672 referred of not being informed about U = U.

Overall, 334 patients (50%) referred that being undetectable did not help them in communicating their status to others, including their GPs.

Significant differences regarding the perceived stigma appeared related to higher level of education, residency in Southern Italy and married marital status, as shown in **Table 3**.

**Table 3:** Patient’s subjective perception of the disease according to socio-demographic variables.

	*“Yes”*	*“No”*	*“I don’t know”*	*P value*
**Sex**				
Males (N = 462)	128 (28%)	226 (49%)	108 (23%)	*p = 0*.*841*
Females (N = 210)	54 (26%)	108 (51%)	48 (23%)	
**Age**				
<50years (N = 293)	79 (27%)	153 (52%)	61 (21%)	*p = 0*.*478*
>50years (N = 379)	103 (27%)	181 (48%)	95 (25%)	
**Education level**				
Elementary license or inferior (N = 285)	66 (23%)	137 (48%)	82 (29%)	***p = 0*.*007***
High school license or superior (N = 387)	116 (30%)	**197 (51%)**	74 (19%)	
**Geographical position**				
Northern Italy** (N = 246)	91 (37%)	114 (46%)	41 (17%)	***p<0*.*001***
Southern Italy° (N = 426)	91 (21%)	**220 (52%)**	115 (27%)	
**Marital status**				
Married (N = 241)	45 (19%)	**125 (52%)**	71 (29%)	**p = 0.003**
Not married (N = 431)	167 (32%)	109 (48%)	85 (20%)	
**Time of diagnosis**				
<10years (N = 337)	102 (30%)	164 (49%)	71 (21%)	p = 0.134
>10years (N = 335)	80 (24%)	170 (51%)	85 (25%)	

*Northern Italy: Florence, Genoa

** Southern Italy = Bari, Foggia.

No significant difference was found for age, sex and duration of disease for the considered query.

### Management of Drug- drug interactions (DDIs) and comedications

**Fig 2:**
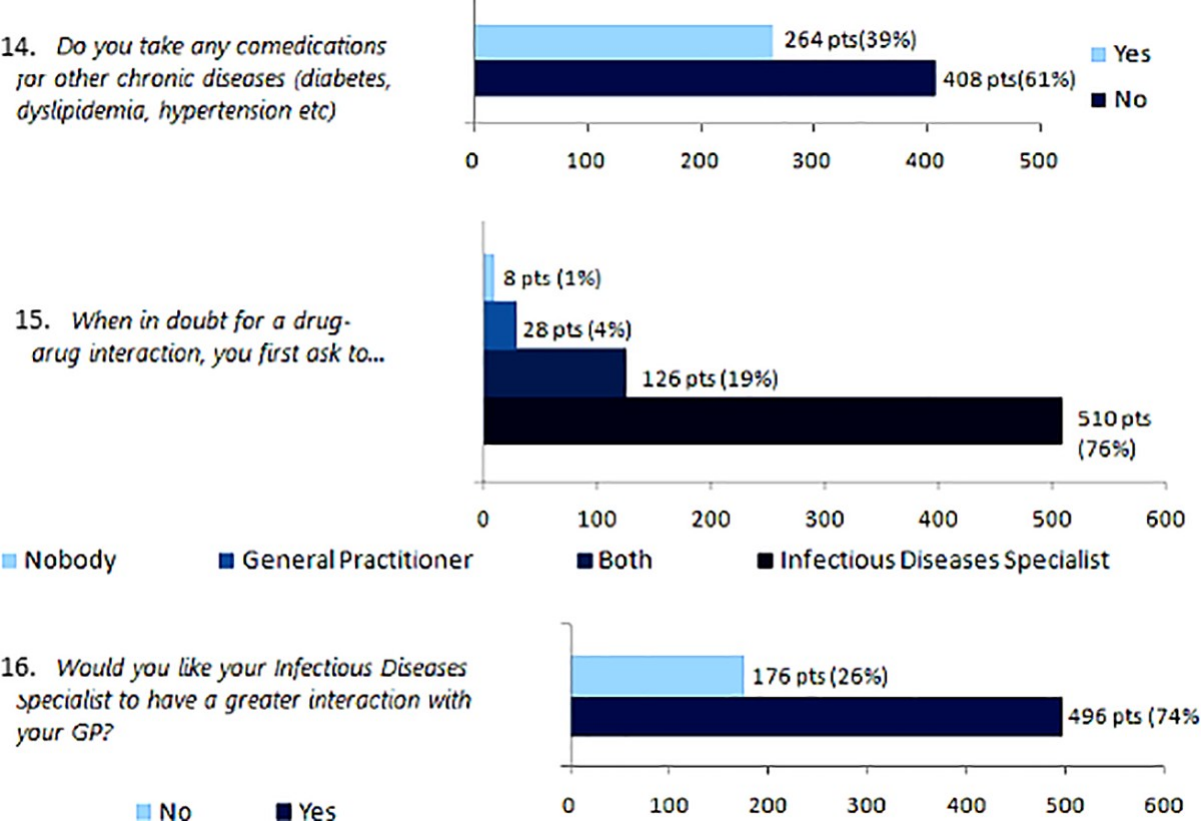
Management of co-medications and drug-drug interactions. Last three items of the survey investigated about the presence and the management of comedications.

Last three items investigated the presence of co medications and their management (Fig 2).

264 patients (39%) assumed other drugs.

Overall, 510 subjects (76%) consulted the ID Specialist for potential DDIs.

Approximatively the 74% of the total population (n = 496) advised a better communication between the GP and the ID Specialist.

## Discussion

As a consequence of the high efficacy and diffusion of cART, life expectancy for patients with chronic HIV infection has significantly improved [1].

As predictable, the incidence of age-related non-communicable diseases in this population has markedly increased, leading ID Specialist to face clinical challenges often far from HIV-related issues [8,17].

At the moment of writing, HIV care in Italy is totally in charge to 5,471 hospital-centered ID Specialists covering 141 Infectious Diseases Units and is completely reimbursed by the National Healthcare System [18]; in accordance to last HIV-care national guidelines [19], PLWHIV under cART with stable undetectable viral load and satisfying immunological condition, receive in media one specialist visit and one blood test every three months.

Nevertheless, numerous social, clinical and geographical factors have been described that can reduce the adherence to the continuum of care process [20], which has been furtherly complicated by the explosion of Sars-COV-2 pandemic, that has enormously enhanced the already high pressure on central Hospital structures and on Infectious Diseases Units in particular. [21].

For all these reasons, the management of PLWHIV needs to be urgently re-written in a new, de-centralized, multidisciplinary view.

In this scenario, General Practitioners (GPs) could represent potential partners, in consideration of their expertise in the management chronic non-communicable diseases, of their human closeness to patients and their families but above all, for the capillary distribution of the General Medicine service on the Italian territory.

Developing potential collaboration models between GPs and specialists has been the focus of many collaborative care projects in diverse fields of Medicine over the last decades and a number of studies have collected providers opinions about this issue on both sides, mainly with a quantitative approach [22–24]. Lack of time, poor financial compensation and a subtle prejudice towards other professional’s knowledge, appeared as the major concerns hindering the construction of a solid relationship between Specialist and GPs.

Similarly, moving to the field of HIV care, the interviewed-structured experience of King et al. performed among GPs, outlined that a strategy to enhance the cooperation between GPs and specialists was strongly needed already twenty years ago, especially in geographical districts at high-prevalence of HIV infection, but was at a time complicated by lack of expertise and time, inadequate communication with specialist services and concern about confidentiality [12].

Surprisingly, though, studies investigating patients’ perspectives about a collaboration between specialists and GPs are scarce and mostly date back to the beginning of AIDS pandemic.

Fear of discrimination, concern about poor confidentiality, sympathy and expertise highly limited patients with HIV infection to tell their GP about their condition, in a era when HIV diagnosis inevitably led to a negative prognosis and to heavy social stigma [14–16].

Still in 2000s, a similar scenario emerged from a depth survey-study published by Petchey et al [24], describing PLWHIV as reluctant to involve their general practitioner (GP) in their care.

Today, the world of HIV has been totally changed by the spreading of modern cART. Nevertheless, changes into patients’ perspectives have never been revalued, at least in Italy.

We therefore choose to collect patients’ opinions to understand their level of communication with their GPs as we believe that, especially for people affected by HIV infection, a full confidence in the healthcare provider should be the starting point for the construction of any form of solid health-care relationship.

In contrast with the above mentioned past experiences our experience outlined the existence of an overall good level of communication between PLWHIV and GPs, in particular in patients with specific socio-demographic features (old age, long history of HIV disease, low level of education).

Nevertheless, fear for social stigma and privacy violation were still complained by a remarkable proportion of patients, that identified these negative feelings as a barrier towards the construction of a healthy patient-doctor relationship.

Another interesting finding regarded the knowledge of the Undetectable = Untrasmittable equation [25–27].

Unexpectedly, one person out of four revealed to ignore what reaching an undetectable viral load meant for their health: this observation brings out how much work is still left for HIV specialists to do to spread the information around the elimination of HIV transmission risk with cART assumption.

Moreover, patients with proper level of information stated that the achievement of stable undetectable viral load did not help them to feel more comfortable to speak about their condition, not even with their GPs: this response was taken as an indirect measure of elevated stigma perception.

Sense of social discrimination was heavier in well-educated patients (highlighting the complexity of the feeling of social discrimination that could be even worst in people with solid intellectual tools and correct level of information), married patients (probably because of a higher feeling of social and familiar responsibility in those subjects) and patients living in South Italy.

Profound contrasts were outlined comparing patients’ feedbacks collected in Florence and Genoa, rather than in Bari and Foggia.

Reasons behind this phenomenon could be related to the existence of social, political and economic determinants that historically separate the Northern and the Southern part of the Italian country; a prompt evaluation of these factors is required in order to improve as much as possible the quality of the health care service, regardless of the patient geographical position.

As expected, over the half of patients participating the survey was over 50 and, for a large part, assumed co-medications.

The ID specialist was the reference point for co-medications management for almost all the interviewed patients, who often complained about their GPs unfamiliarity with ART and DDIs.

This feedback highlights how mutual trust and collaboration still need to be built from the ground up, not only between GPs and ID Specialist, but firstly between GPs and PLWHIV.

### Limitations

This study has some methodological limitations.

Firstly, the questionnaire tool proposed has been self-developed by co-authors, because no existing questionnaire, even if developed for HIV patients [28,29], followed a shared validation process neither could fit in the aim of this study.

Moreover, as questions specifically addressed HIV-positive population and topics related to the perception and the communication of their condition, proposing the questionnaire to a control group (i.e. HIV-negative patients, or hospital personnel) to check the reliability of answers, would have not make sense and could possibly have added individual confounders. For this reason, face validity and internal consistency have been assessed by performing accompanied pilot-interviews on a small number of respondents, as explained in Methods section, but the survey does not follow a validated scale.

The second major limitation was that no sample size calculation was planned for this survey, but a six-months-time frame (from December 1^st^, 2019 to May 31^st^, 2020) was set, during which all patients consecutively attending the participating centers would have been interviewed.

Nevertheless, because of the discontinuation of the out-patients services in all the country started with the explosion of Sars-COV-2 pandemic, data collection was stopped at the beginning of February 2020.

Should be also be mentioned that, due to the nature of this survey (anonymous, self-compiled, filled on a complete voluntary basis), it was impossible to precisely establish the rate of attendance, as the proportion of patients refusing to participate was not recorded.

Unfortunately, our questionnaire was available only in Italian language and this has inevitably limited the participation to the survey of all non- Italian- speaking patients; their opinions could have changed the presented results and deserve further investigation, also considering that migrants represent a remarkable proportion of PLWHIV, especially in Southern Italy.

A wider, multilanguage, distribution of similar interviews is therefore advisable, possibly extended to a larger number of Infectious Diseases Centers to collect a more accurate perspective of patients’ opinions on the Italian territory.

## Conclusions

According to the preliminary results of this survey, great help could come from GPs as partners for ID Specialists in caring for chronic HIV infection.

Nevertheless, fear of social exclusion and privacy violation, along with poor trust in GPs familiarity with cART management, persists as crucial points to work on, potentially impairing the relationship between GPs and PLWHIV.

## Supporting information

S1 Table 16-items questionnaire proposed to PLWHIV–English translation of the survey tool. (PDF)

S2 Table 16-items questionnaire proposed to PLWHIV–original version of the survey tool in Italian language. (PDF)
