# Supplementary material for: General Practitioners as partners for a shared management of chronic HIV infection: An insight into the perspectives of Italian People Living with HIV
Source: PLoS One. 2021 Jul 9;16(7):e0254404. doi: 10.1371/journal.pone.0254404 (PMC8270424; doi:10.1371/journal.pone.0254404)
Supplement: S1 Table — (PDF) [file pone.0254404.s001.pdf]

Clinic: ____________________

1. Sex
- ☐ Male
- ☐ Female

2. Residence ______________________________

3. Age
- ☐ 18 – 29 years
- ☐ 30– 49 years
- ☐ 50 – 69 years
- ☐ >70 years

4. Nationality
- ☐ Italian
- ☐ Non Italian: _______________________

5. School license
- ☐ None
- ☐ Elementary license
- ☐ High school license
- ☐ Graduation or superior

6. Marital status
- ☐ Married
- ☐ Living with parents
- ☐ Living with coabithee
- ☐ Single

7. *How long have you been diagnosed with HIV?*
- ☐ < 5years
- ☐ 5 – 15years
- ☐ > 15years

8. *Have you informed your General Practitioner about HIV positivity status?*
- ☐ Yes
- ☐ No

9. *If not, why?*
- ☐ I am afraid that my family could be informed about my diagnosis
- ☐ I think that is not necessary to made him aware of my status
- ☐ I am scared of being discriminated

10. *If not, are you worried about the fact that you doctor misses this part of your medical history?*
- ☐ Yes
- ☐ No

11. *If not, do you think that informing your General Practitioner about your status would improve your global quality of life ?*
- ☐ Yes
- ☐ No

12. *Is your General Practitioner informed about your antiretroviral medications?*
- ☐ Yes
- ☐ No

13. *"Undetectable= Untransmittable".*
Does being undetectable help you in communicating your status to your General Practitioner and/or to others?
- ☐ Yes
- ☐ No
- ☐ I don't know

14. *Do you take any co-medications for other chronic diseases (diabetes, dyslipidemia, hypertension exc.)?*
- ☐ Yes
- ☐ No

15. *When in doubt for a drug-drug- interaction, you first ask to…*
- ☐ Infectious Diseases Specialist
- ☐ General Practitioner
- ☐ Both
- ☐ Nobody

16. *Would you like your General Practitioner to have a greater interaction with your Infectious Diseases Specialist?*
- ☐ Yes
- ☐ No
