# Supplementary material for: General Practitioners as partners for a shared management of chronic HIV infection: An insight into the perspectives of Italian People Living with HIV
Source: PLoS One. 2021 Jul 9;16(7):e0254404. doi: 10.1371/journal.pone.0254404 (PMC8270424; doi:10.1371/journal.pone.0254404)
Supplement: S2 Table — (PDF) [file pone.0254404.s002.pdf]

Centro: ____________________

1. Sesso
- ☐ Maschio
- ☐ Femmina

2. Residenza ____________________________

3. Età
- ☐ 18 – 29 anni
- ☐ 30– 49 anni
- ☐ 50 – 69 anni
- ☐ >70 anni

4. Nazionalità
- ☐ Italiana
- ☐ Straniera (indicare quale): ______________________

5. Titolo di studio
- ☐ Nessuno
- ☐ Licenza elementare
- ☐ Diploma
- ☐ Laurea o superiore

6. Situazione abitativa
- ☐ Vivo con moglie/figli/entrambi
- ☐ Vivo con i genitori
- ☐ Vivo con covivente
- ☐ Vivo solo

7. *Da quanti anni sa di essere positivo?*
- ☐ < 5 anni
- ☐ 5 – 15 anni
- ☐ > 15 anni

8. *Ha comunicato al Suo Medico di Famiglia di essere sieropositivo?*
- ☐ Si
- ☐ No

9. *Se no, per quale motivo principale?*
- ☐ Temo che possa divulgare informazioni sul mio stato alla mia famiglia o ad altri
- ☐ Credo non sia necessario comunicargli la mia patologia
- ☐ Temo di essere discriminato

10. *Se no, la preoccupa l'idea che il suo medico ignori parte della sua storia clinica?*
- ☐ Si
- ☐ No

11. *Se no, ritiene che condividere con il medico la notizia della sua sieropositività migliorerebbe la sua qualità della vita complessiva?*
- ☐ Si
- ☐ No

12. *Il suo Medico di Famiglia è a conoscenza della terapia antiretrovirale che assume?*
- ☐ Si
- ☐ No

13. *"Undetectable= Untransmittable".*
Essere stabilmente con caria virale negativa potrebbe facilitare la comunicazione della sieropositività al suo medico o ad altre persone?
- ☐ Si
- ☐ No
- ☐ Non so

14. *Assume terapie per altre patologie croniche (diabete, ipertensione, dislipidemia etc…)?*
- ☐ Yes
- ☐ No

15. *A chi si rivolge per informazioni sulle possibili interazioni tra le terapie che assume?*
- ☐ Infettivologo
- ☐ Medico di Famiglia
- ☐ Entrambi
- ☐ Nessuno dei due

16. *Secondo lei, la comunicazione tra il suo Infettivologo e il suo Medico di Famiglia andrebbe potenziata?*
  - ☐ Si
  - ☐ No
